# Co-selection for antibiotic resistance by environmental contaminants

**DOI:** 10.1038/s44259-024-00026-7

**Published:** 2024-04-01

**Authors:** Laura May Murray, April Hayes, Jason Snape, Barbara Kasprzyk-Hordern, William Hugo Gaze, Aimee Kaye Murray

**Affiliations:** 1 European Centre for Environment and Human Health, University of Exeter Medical School, Faculty of Health and Life Sciences, Environment and Sustainability Institute, Penryn, Cornwall UK; 2 Formerly AstraZeneca Global Environment, Alderley Park, Macclesfield, UK; 3 Department of Chemistry, University of Bath, Bath, UK

**Keywords:** Antimicrobial resistance, Antibiotics, Environmental microbiology

## Abstract

The environment is increasingly recognised as a hotspot for the selection and dissemination of antibiotic resistant bacteria and antibiotic resistance genes. These can be selected for by antibiotics and non-antibiotic agents (such as metals and biocides), with the evidence to support this well established by observational and experimental studies. However, there is emerging evidence to suggest that plant protection products (such as herbicides), and non-antibiotic drugs (such as chemotherapeutic agents), can also co-select for antibiotic resistance. This review aims to provide an overview of four classes of non-antibiotic agents (metals, biocides, plant protection products, and non-antibiotic drugs) and how they may co-select for antibiotic resistance, with a particular focus on the environment. It also aims to identify key knowledge gaps that should be addressed in future work, to better understand these potential co-selective agents.

## Introduction

Antimicrobials are agents which kill or inhibit the growth of microorganisms (bacteria, fungi, viruses, and parasites)^1^. Antimicrobial resistance (AMR) occurs when the organisms these agents target evolve to survive their toxic effects. AMR is a global health concern, with predictions that by 2050 it could be responsible for 10 million deaths per year^2^. Antibiotics are a subclass of antimicrobials, that are used to target bacterial infections in humans, animals, and plants. Resistance of bacteria to antibiotics is termed ‘antibiotic resistance’, and is of significant concern since antibiotics are used frequently in medicine to treat bacterial infections, e.g., tuberculosis^3^, and as prophylaxis, e.g., before major surgery^4^. In 2019, 4.95 million deaths were associated with drug resistant bacterial infections, of which 1.27 million deaths were directly attributed to drug resistant bacterial infections^5^. Antibiotic resistance can be acquired through *de novo* mutation, or through horizontal gene transfer (HGT) of antibiotic resistance genes (ARGs) encoded on mobile DNA elements (such as plasmids, integrons or transposons) that are passed between bacterial cells of the same or different species^6^. HGT rate can change depending on various factors such as exposure to stressors (e.g., antibiotics), changes in pH, and oxidative stress^6^, therefore, additional anthropological input of environmental contaminants that can increase HGT are of concern to environmental and human health.

Strategies to address antibiotic resistance have included attempting to reduce selection pressure by decreasing clinical and veterinary antibiotic consumption^7^. However, antibiotics are not the only agents capable of contributing to antibiotic resistance evolution. Agents other than antibiotics have antimicrobial properties, including biocides (e.g., quaternary ammonium compounds (QACs)) and metals (e.g., copper, zinc). These agents can indirectly select for antibiotic resistance and ARGs^8–10^, through a process known as co-selection. Consequently, exposure to these agents may increase resistance to antibiotic drugs and other antimicrobial compounds, even in the absence of antibiotic selective pressure.

**Fig. 1:**
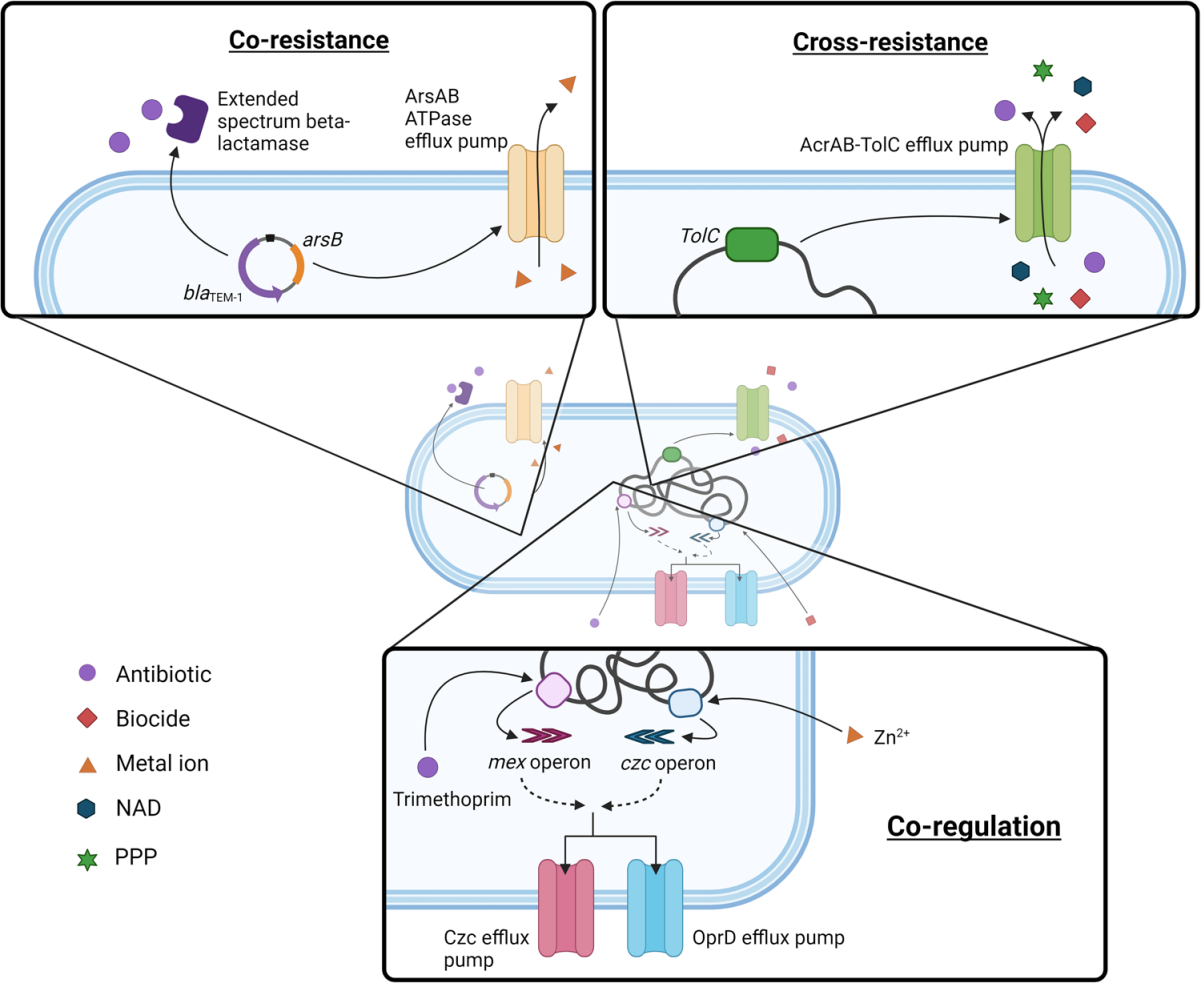
Co-selection mechanisms of cross-resistance, co-resistance and co-regulation. Cross-resistance occurs when a mechanism provides resistance to two or more agents (e.g., a multi-drug efflux pump). In this figure, the AcrAB-TolC efflux pump gene is shown, which can pump out of the cell multiple antibiotics and biocides^137^, non-antibiotic drugs^86,138^, and plant protection products^32,139^. Co-resistance occurs when two genes are physically linked on a piece of DNA so are inherited together e.g., an antibiotic and metal resistance gene located on a plasmid. In this figure, the metal resistance gene *arsB* and the antibiotic resistance gene *blaTEM-1* are both present on a plasmid and can be inherited together^139^. Co-regulation occurs when the translational or transcriptional responses to one agent leads to a co-ordinated response to more than one agent (e.g., an antibiotic or biocide could lead to expression of a multi-drug efflux pump). In this figure, the transcriptional pathways of the *mex* operon and the *czc* operon are linked so that expression of either leads to the expression of both the *czc* and oprD efflux pumps^140^. Created with Biorender.

Co-selection refers to the simultaneous selection for resistance to multiple agents and can occur through three different processes: co-resistance, cross-resistance and co-regulation (Fig. 1). Co-resistance occurs when multiple resistance genes are genetically linked, for example, on a conjugative plasmid^8,9^. Cross-resistance occurs when one mechanism provides resistance to more than one agent, for example, a multi-drug efflux pump removing more than one agent from the cell^8,9^. Co-regulation occurs when translational and transcriptional responses are linked and produce a co-ordinated response, such as the expression of multiple separate efflux pumps, triggered by the presence of one agent^9^. Co-selection has been acknowledged as a key mechanism that selects for ARGs in different microbial communities.

## The relevance of the environment

The environment is increasingly recognised as an important reservoir of antibiotic resistance in which ARGs may spread between bacteria and be potentially selected for by micropollutants^11–13^. Antibiotic resistant bacteria in the environment and the genes they carry can then be transmitted to humans through food, drinking water, air, or through direct contact with the environment, such as through recreational use of coastal waters^14^.

Selection and co-selection can occur over wide concentration ranges of selective agents, illustrating the importance of the environment, since concentrations of antibiotics and other non-antibiotic agents can vary by orders of magnitude^15–17^. Clinical breakpoints are defined using determination of the Minimum Inhibitory Concentration (MIC), which is the lowest drug concentration where growth is inhibited^18^. Organisms that grow in concentrations of antibiotic higher than the MIC are considered resistant. However, subinhibitory concentrations can amplify resistance or increase persistence of resistance. The Minimal Selective Concentration (MSC) is the lowest concentration of an agent where the growth of resistant and sensitive strains is equal, and concentrations between the MSC and MIC of the resistant strain positively select for (i.e., amplify) resistant strains^19^. The Minimal Increased Persistence Concentration (MIPC) indicates the antibiotic threshold at which the rate of loss of the resistant strain is significantly slowed, compared to when no selective pressure is present. Concentrations between the MIPC and MSC lead to prolonged maintenance of resistant strains, even though there is no positive selection or amplification occurring^20^. Antibiotics and other potentially co-selective agents are present in human, animal, and environmental microbiomes at these sub-MIC concentrations, particularly in the environment where they are found within the ng-g/L range^15^, resulting in a large temporal and geographical antibiotic resistance selection window. There is evidence to suggest that antibiotic resistance can be selected for and/or maintained in the environment, due to selective agents that are present in municipal, industrial, and agricultural pollution^21–25^.

This non-systematic review aims to provide an overview of the co-selective effects of biocides, metals, non-antibiotic drugs (NADs), and plant protection products (PPPs), with a particular focus on the environment, all within a single paper. Previous reviews have covered some of these agents individually and we also direct the reader to these (e.g., for metals^9,26,27^; for biocides^28,29^; for NADs^30,31^; and for PPPs^32^). The review also aims to highlight some of the important concerns and knowledge gaps that have been identified with regards to these agents in the understanding of AMR evolution and dissemination in an environmental context. Suggestions as to how future research might address these points are also provided.

## Metals

Metals naturally occur in the environment, with elevated concentrations resulting from pollution caused by anthropogenic activity (e.g., historical and current mining activity)^33^. Metals are utilised by humans for numerous purposes in different settings, including disinfection in human healthcare^34^; antifouling agents in aquaculture^35^; feed additives in animal husbandry^36^ and crop protection in agricultural practices^37^. The presence of metals is often beneficial to microorganisms as they are used as micronutrients and are required for survival^38^. However, some metals are toxic to bacteria and even essential metals can become toxic at high concentrations, or can inhibit bacterial growth^39^. Metals can select for chromosomal and plasmid-borne resistance mechanisms that ameliorate the toxic effects of metals in bacterial cells. These metal resistance mechanisms can also be genetically linked to antibiotic resistance genes (i.e., co-resistance)^10,40^; may share their mechanism of resistance with antibiotics (cross-resistance)^41^; or can be expressed alongside antibiotic resistance genes as a result of linked regulatory systems (co-regulation)^42^.

Metal presence and contamination is ubiquitous^33^, so co-selection for antibiotic resistance could occur in a wide variety of environments including wastewater, freshwater, manures, and soils, depending on bioavailability. The bioavailability of metals can be affected by factors including sorption to soil or sediment particles, pH, changes to ionic composition, or redox potential^43^. For these reasons, the presence of metals in some environments could result in differing risk of selection for ARGs than in others. For example, environments receiving mine waste are likely to have increased concentrations of metals, and therefore may have an increased selective pressure^44^. Other environments, e.g., manures and wastewater^15^, are more likely to harbour a range of pollutants including antibiotics, ARGs, and antibiotic resistant bacteria, which may increase risk of selection for antibiotic resistance through co-occurrence of multiple selective pressures and the genes/organisms upon which they may act. Concerns have long been expressed about the contribution of metals to the proliferation of antibiotic resistance via indirect selection pressures^9^, particularly as their stability may lead to long term selective pressures within a large temporal window^13,45,46^.

Numerous studies have investigated the relationship between antibiotic and metal resistance, and the topic is well reviewed elsewhere^9,26,27^. Studies that support co-selection for antibiotic resistance by metals have been conducted experimentally, in vitro using single species and complex communities of bacteria, and in situ in various environmental compartments including sewage sludge, aquaculture, and agricultural soils^9,26,27^. There is also evidence of co-selection for antibiotic resistance at sub-inhibitory concentrations of metals^47–51^.

It is well established that antibiotic and metal resistance genes can co-occur within bacterial genomes and this has been explored in genomic studies which use publicly available, fully sequenced genome data from hundreds of bacterial species^10,40^. These co-occurrence patterns can arise as a result of the resistance genes simply co-existing within the bacterial cell (e.g., the metal resistance gene present on the chromosome and the antibiotic resistance gene on a plasmid), which one study found to be the most common route of co-occurrence^10^. Perhaps more alarmingly, although less common, the metal and antibiotic resistance genes can co-occur on mobile genetic elements such as plasmids^10,40^, and would lead to co-resistance under selective pressure by either agent but also potential HGT of both types of resistance. An example of this is bacitracin resistance genes, which can co-occur on plasmids with copper and zinc resistance genes^40^. Interestingly, plasmids that harbour metal resistance genes have been shown to be more likely to also contain ARGs than plasmids which do not carry metal resistance genes, and these plasmids were also more likely to be conjugative^10^. These genomic studies have shown several ARG classes are more likely to be associated with metal resistance genes, including beta-lactam, kasugamycin, bacitracin, aminoglycoside, polymyxin, and tetracycline resistance genes^40^. The most common metal or antibiotic resistance genes detected in these genomic based studies are often related to those agents which are most frequently used^40^, likely owed to a greater selection pressure and therefore a greater need for bacteria to evolve, maintain and disseminate resistance genes.

The evidence for co-selection for antibiotic resistance by metals is vast, however, less is known about how this translates to selection for antibiotic resistance in the environment, and further research is required to understand the selective windows across which metals can act. This is of particular concern since metals are highly persistent in the environment, suggesting that there could be a much larger temporal co-selective window than for other non-antibiotic agents.

## Biocides

Biocides are defined by the European Commission as ‘any substance or mixture’ ‘with the intention of destroying, deterring, rendering harmless, preventing the action of, or otherwise exerting a controlling effect on, any harmful organism by any means other than mere physical or mechanical action’ (Regulation (EU) No 528/2012). This definition includes antibiotics, disinfectants, herbicides, pesticides, and other related compounds. For the purposes of this review, biocides used as disinfectants and detergents (such as quaternary ammonium compounds, ‘QACs’) are covered here, and biocides used in other circumstances are included elsewhere in this review.

The application concentration of biocides is many times higher than the MIC, and in-use concentrations are often thousands of times greater than the MIC, often in the g/L range^52^. However, they are also found in the environment at lower, sub-lethal concentrations (µg/L) following dilution or can be found as residues on many surfaces. These sub-lethal concentrations may select for antibiotic resistance.

There is mixed evidence regarding associations between antibiotic and biocide resistance. Several reviews have explored this topic, with some outlining increases in antibiotic resistance after exposure to disinfectants^8,28,53^, yet others have found no association between exposure to biocides and antibiotic resistance^8,54,55^. For example, one study demonstrated that there was no co-selection (tested by identifying MIC profiles for various antibiotics and disinfectants) between chlorhexidine and the antibiotics tested, in over one hundred S*almonella* spp. isolates^56^. Whereas a later study showed that when *Klebsiella* spp. were exposed to chlorhexidine, resistance to colistin was co-selected for (with the increased expression of AMR genes, and a multi-drug efflux pump)^57^.

However, like metal resistance genes, biocide resistance genes are more likely to be genetically associated with ARGs, and are therefore likely to be co-selected for via co-resistance. Analysis of plasmid data from publicly available genome data has suggested that bacteria carrying plasmids with biocide resistance genes are more likely to also carry ARGs than those not carrying these plasmids^10^. QAC resistance genes have also been found on plasmids containing metal, beta-lactamase, trimethoprim, or aminoglycoside resistance genes, and are also found in conserved regions of integrons in both Gram-positive and Gram-negative bacteria^8^. The gene *qacEΔ1* which confers resistance to several biocides, including QACs^10^, is found on class 1 integrons^58^ and Tn21 transposons^59^. These genetic data suggest that biocides, including QACs, have the potential to co-select for antibiotic resistance via co-resistance. Furthermore, the mobilisation potential of these genes provides an increased likelihood of this resistance passing between environmental and clinically relevant bacterial strains.

Genetic linkage of biocide resistance genes and ARGs does not confirm that co-selection has occurred. However, experiments in vitro have also demonstrated co-selection for antibiotic resistance by biocides in both single species experiments, and in bacterial communities. For example, an aquatic bacterial community exposed to benzalkonium chloride increased resistance to ciprofloxacin and penicillin G, through cross-resistance^60^. Exposure of *Escherichia coli* to 0.2 mg/L triclosan for 30 days increased mutation frequency, and antibiotic resistance, compared to non-exposed cells^61^. Exposure of *E. coli* to sodium chlorite and iodoacetic acid for 40 subculture cycles of 40 days increased resistance to amoxicillin and ciprofloxacin to ‘clinically relevant’ levels via upregulation of multidrug resistance efflux pumps associated with antibiotic resistance^61,62^. These demonstrate that co-selective effects of biocides can be detected both in single species, and in community studies.

However, other studies show biocides may not always co-select for antibiotic resistance. When a wastewater influent bacterial community was exposed to 8 mg/L benzalkonium chloride, there were no identifiable increases in ARG prevalence, but there were changes to the community composition according to reads-based metagenome analyses^63^. This may suggest that only intrinsically resistant species were enriched, that changes in regulation of genes involved in antibiotic resistance occurred which could not be detected with the approach used, or alternatively, that any genes selected for were not present in gene databases used for these analyses, or that there was simply no co-selective effect in this model community. The databases used to identify ARGs focus on clinically important strains and will therefore lack resistance genes that confer low-level resistance, or genes more prevalent in environmental strains. A recent functional metagenomic study illustrated that cross-resistance to biocides and antibiotics can be mediated by metabolic genes^64^, which would not be present on commonly used ARG databases.

In summary, in certain circumstances, exposure to biocides may increase resistance to some antibiotics, but it is not clear to what extent these effects may be consistent across all environments, whether these genes are stably heritable, and whether they confer high levels of resistance that would be of clinical concern. Additionally, different environments can contain large concentration ranges of biocides, often alongside antibiotics, and the consequences of co-occurrence are largely unknown. Further research is needed to understand these effects, for example, by testing a large concentration range (from point of use to residual and micropollutant concentrations). Additionally, longer-term experimental studies looking at both the selection for *de novo* mutants, and the sustained selection of these over time, are required. This is considered in more detail in the Discussion.

## Non-antibiotic drugs (NADs)

NADs, for the purposes of this review, refer to any pharmaceutical used in human and veterinary medicine that is not an antibiotic. These compounds can be found at various concentrations in the human body and are also found at low concentrations in the aquatic environment^65^. There is increasing interest in some NADs as antibiotic adjuvants^66,67^. However, there is also increasing interest in their potential to co-select for antibiotic resistance.

There are several studies investigating reduction in growth by NADs with single species models^68–72^. One of these studies^71^ indicated that the AcrAB-TolC efflux pump was important in allowing the growth of *E. coli* when under stress of the NADs tested, since knockout mutants had inhibited growth compared to wildtype. Reduction in growth with antibiotics can be a good proxy for identifying MSCs for antibiotic resistance^63,73^. However, one study showed that whilst some chemotherapeutic agents reduced the growth of *E. coli* and *Staphylococcus aureus*, there was no corresponding increase in MIC, suggesting that there was no co-selective benefit^74^. Therefore, although reduction in growth could indicate co-selective effects, this cannot be confirmed without further experimental work.

Several NADs have also been shown to increase HGT rates between bacteria. Ibuprofen, diclofenac, naproxen, propranolol, and gemfibrozil can increase the rate of transformation (the acquisition of free DNA from the environment into bacterial cells) of a plasmid bearing ARGs in *Acinetobacter baylyi*^75^. Carbamazepine and acetaminophen, as well as some antidepressants, can increase conjugative transfer in *E. coli*^76,77^ which may allow for acquisition of plasmids bearing ARGs and genes involved in NAD resistance, or plasmids containing ARGs alone. Carbamazepine was shown to increase conjugation rates at environmental (0.05 mg/L) concentrations, raising concerns that there could be a large spatial window over which co-selection by this compound could occur^78^. Therefore, some NADs may promote the spread of AMR, even if they do not co-select for antibiotic resistance themselves. However, this may be pharmaceutical specific, since some anti-HIV drugs reduced the rate of ARG transfer through conjugation in *E. coli* and *Klebsiella pneumoniae*^79^.

Additionally, some studies have investigated phenotypic resistance to antibiotics after exposure to NADs. One study showed that *E. coli* exposed to 5–100 mg/L of the antidepressant fluoxetine for 30 days exhibited increased antibiotic resistance to chloramphenicol, amoxicillin, and tetracycline, which may have been mediated by increased upregulation of multidrug resistant efflux pumps^80^. Fluoxetine also increased the mutation rate in *E. coli*, via ROS-mediated mutagenesis^80^. The chemotherapeutic drug methotrexate also co-selected for trimethoprim resistance at concentrations 40 to 320 fold lower than its MIC, with MSCs experimentally determined for chromosomal (200 µg/mL) and plasmid borne (25 µg/mL) trimethoprim resistance using tagged isogenic *E. coli* strains^81^. Similarly, exposure to eight different chemotherapeutic drugs (daunorubicin, epirubicin, mitomycin C, gemcitabine, bleomycin, dacarbazine, and azacitidine) at therapeutic concentrations increased mutation rates in *E. coli* through increased activation of the SOS response^82^. Additionally, this same study found that three chemotherapeutic drugs selected for imipenem resistant *Pseudomonas aeruginosa*, ciprofloxacin resistant *S. aureus*, and cefotaxime resistant *Enterobacteria cloacae*^82^. The non-steroidal anti-inflammatory drug diclofenac at 80 µg/L has been shown to lead to changes in gene expression, leading to increases, and also decreases in phenotypic resistance to antibiotics in *S. aureus*^83^. Diclofenac led to increased resistance to oxacillin and vancomycin, decreased resistance for ciprofloxacin, orfloxacin, and norfloxacin, but had no effect on susceptibility to tetracycline or chloramphenicol, indicating that collateral sensitivity may limit extensive multi-drug resistance acquisition after exposure to diclofenac, or other NADs^83^. The atypical antipsychotic medication quetiapine, at concentrations likely to occur in the human gut, has been shown to increase the expression of *marA*, *acrA*, and *tolC*, and can reduce *ompF* expression, also increasing antibiotic resistance in *E. coli*^84^. Finally, exposure to the antidepressants amitriptyline, fluoxetine, and sertraline can increase phenotypic antibiotic resistance in *Acinetobacter baumanni*^85^.

A large co-selective effect of NADs may be through cross-resistance, through increased expression of efflux pumps. For example, diclofenac has been shown to be a substrate of the multi-drug efflux pump AcrAB-TolC^86^. Although not tested, exposure to these NADs may increase expression of AcrAB-TolC and increase resistance to antibiotics. This could be a target for future research.

These studies (and most studies testing NADs) often use concentrations that are much higher than those present in the environment. However, concentrations of NADs present in hospital and municipal wastewater treatment plants have also been shown to increase ciprofloxacin resistance in *Salmonella enterica* serotype *Typhimurium* by increasing mutation rates^87^. Therefore, there is some evidence to suggest that effects seen at high concentrations may also occur at lower concentrations, suggesting selection could occur in wastewater, or potentially freshwater. However, there is some conflicting evidence, with a recent publication^88^, indicating that 30 day exposure of *E. coli* to acetaminophen, ibuprofen, TiO2, metformin, and propranolol at environmentally relevant concentrations did not lead to increased phenotypic resistance to antibiotics, nor to increases in ARGs.

Finally, there is emerging research testing mixtures of antibiotics and NADs, although there are few studies specifically investigating this. One recent study has shown that *E. coli* exposed to duloxetine and chloramphenicol had a synergistic response compared to exposure to one of these agents alone, with the *E. coli* becoming resistant eight antibiotics, with increased expression of *acrA, acrB* and *marA* (genes involved in efflux pumps)^30^. Most research focuses on mixtures as adjuvants and use growth reduction as an endpoint to show efficacy for this application^67,89–91^. There is a large knowledge gap in how combinations of pharmaceuticals can select for antibiotic resistance, particularly at sub-MIC concentrations.

In summary, there is potential for NADs not only to have antibacterial effects, but to have mechanisms of activity (such as upregulation of efflux pumps, i.e., cross-resistance and/or co-regulation) that overlap those of antibiotics. Thus, any prolonged exposure to these drugs may lead to co-selection for ARGs, though some studies (e.g., ^79^) have shown this may not always be the case. As several of these compounds can increase rates of HGT, there is also the opportunity for an increase in ARG transfer within microbiomes. Research is needed on the effects of NADs on gene expression, such as whether they induce a specific expression profile, lead to co-regulation, or induce mutations that confer cross-resistance (e.g., through upregulation of the SOS pathway^92^). Additionally, since most research into NADs has focused on the human health effects (e.g., on the gut microbiome) and benefits of using NADs as adjuvants, further work is needed to investigate if NADs can also co-select for antibiotic resistance at a range of concentrations relevant to different contexts.

## Plant protection products (PPPs)

PPPs, also referred to as pesticides, are any agent used in agriculture/horticulture that prevent or treat infection or infestation by unwanted organisms. They include herbicides, fungicides (some of which have the same mechanism of action as clinical fungicides^93^), and insecticides, which target weeds, fungi, and insects, respectively. They are biologically active ingredients which are applied in commercial formulations that contain a variety of other ingredients to increase the efficacy of the active chemical^94^.

Increasing human population growth has resulted in higher demands for food and as a result, increased reliance on PPPs for food security. This trend is likely to continue, yet in 2012, world usage of the chemicals at the producer level already totalled over 2.72 billion kg^95^. PPPs are applied at high concentrations (often >g/L of active ingredient as calculated from product labels), repeatedly, to crops and soils, exposing the bacteria in the environment to significant quantities of PPPs over large temporal windows. When conducting environmental risk assessments for these agricultural chemicals, limited testing is carried out on microorganisms. Environmental risk assessments only require testing of carbon and nitrogen turnover, at limited PPP concentrations, over a limited time period, and do not consider the potential impacts on community diversity and antibiotic resistance^96,97^. Due to the vast number of target organisms PPPs act upon, and PPP mechanisms of action, this section investigating co-selection by PPPs will be split according to the major groups: herbicides, fungicides, and insecticides.

## Herbicides

A few studies have investigated co-selection for antibiotic resistance by herbicides. One study investigated the effects of herbicide exposure in soil microcosms using phenotypic testing, metagenomics and qPCR^98^. Selective pressures exerted by glyphosate, glufosinate, and dicamba elevated the relative abundance of a range of ARG classes and MGEs in the soil bacterial communities. These increases occurred irrespective of changes to the community, as only glufosinate had a significant effect on community composition. The effects on relative abundance of ARGs and MGEs were observed at agriculturally relevant concentrations of 10 mg/kg^98^, demonstrating increases in ARGs could potentially occur in the field during herbicide application. Furthermore, *de novo* mutations were found in genes linked to multiple antibiotic resistance mechanisms (e.g., efflux pumps, N-acetyltransferases), and conjugation frequency of a multidrug resistance plasmid increased with exposure to the herbicides, thought to be a result of increased cell membrane permeability and up-regulation of stress-related genes^98^. Glyphosate-induced increases in conjugation frequency were also observed in another study which investigated the transfer of a resistance plasmid between donor and recipient *E. coli* species, which was due to upregulation of genes involved in cell membrane permeability and conjugation^99^.

Isolates of *P. aeruginosa* showed increased phenotypic resistance to aztreonam but did not change in resistance to colistin or polymyxin B, after exposure to the widely used herbicide active ingredients atrazine and diuron^100^. Atrazine has also been shown to significantly increase phenotypic resistance to ciprofloxacin, kanamycin, and streptomycin (but not tetracycline) in single species broth microcosm experiments using *E. coli*^101^. The mechanisms of co-selection in these experiments were not identified. In similar experiments, sublethal concentrations of formulations ‘Roundup’ (active ingredient glyphosate), ‘Kamba’ (active ingredient dicamba) and ‘2,4-D’, also resulted in increased phenotypic resistance to ciprofloxacin in both *Salmonella enterica sv. Typhimurium* and *E. coli*^102,103^. However, they, also reported decreases in phenotypic resistance to other antibiotics (collateral sensitivity), along with variable changes in resistance to other antibiotics that were largely dependent on herbicide, bacterial species, and antibiotic^102^. Efflux was shown to play a role in the increased tolerance of *E.coli* to chloramphenicol and kanamycin in the presence of dicamba and glyphosate respectively^102^. Similar variable results were observed in another study by the group using the active ingredients of the herbicides^104^, and multidrug efflux pump AcrAB-TolC was shown to play a role in the changes in phenotypic resistance, suggesting cross-resistance could be involved^104^. The variation in co-selective potential observed in these studies may be due to class specific effects of these herbicides, and their modes of action, particularly on bacteria, which have not been comprehensively characterised as they are not the target organism.

## Fungicides

Fungicide exposure has also been associated with an increased abundance of ARGs, observed using metagenomic sequencing. The active ingredients carbendazim, azoxystrobin, and chlorothalonil increased the abundance of ARGs including *sul1*, *sul2, aadA, tetX2,* and *tet(L)* in microcosm experiments containing greenhouse and mountain soil^105^. These genes are involved in antibiotic target replacement, drug inactivation, and efflux pump mechanisms^106^.

Exposure of a soil community to azoxystrobin at agriculturally relevant concentrations also showed increased community phenotypic antibiotic resistance to aminoglycosides (streptomycin, kanamycin, and gentamicin)^107^, and commercial formulations of azoxystrobin were shown to increase phenotypic resistance to ampicillin, and chloramphenicol, but have no effect on tetracycline resistance in two soil types (loamy sandy soil, and clay loam soil)^108^. An increase in resistance to streptomycin was also observed in the loamy sandy soil, but sensitivity to this antibiotic was observed in the clay loam soil, demonstrating how soil properties may impact response to the fungicide. Again, the mechanisms involved in these increases in resistance were not determined^108^.

In addition to these findings in soil communities, azoxystrobin across a range of concentrations (0.1–5 mg/kg), altered the gut microbiome of the soil-dwelling worm *Enchytraeus crypticus* and increased the relative abundance of unclassified ARGs, analysed by Illumina amplicon sequencing and high-throughput qPCR^109^. However, the authors concluded it was unclear whether the increase in ARGs was a result of the changing gut community, or due to direct selection for ARGs.

There is little research investigating changes in phenotypic resistance to antibiotics after, or with exposure to fungicides in single species experiments. However, there are studies investigating how fungicides may increase HGT of ARG-bearing plasmids^110,111^. The fungicide ‘mancozeb’ was shown to facilitate plasmid mediated ARG transfer in experiments exploring intra-species transfer (*E. coli* donor to *E. coli* recipient) and inter-species transfer (*E. coli* donor to *Pseudomonas putida* recipient), alongside altering the expression of conjugation and stress response genes^110^. In another study that used the same donor and recipient species, the fungicides azoxystrobin and carbendazim enhanced the expression of conjugation related genes, while chlorothalonil was able to enhance reactive oxygen species, activate the stress response and increase membrane permeability, all resulting in the transfer of ARG-containing plasmid RP4^111^.

## Insecticides

To date, insecticides are the least researched of the major PPP groups with regards to co-selection for antibiotic resistance. However, some evidence for potential co-selection exists. Insecticide exposure (‘Pyrethrum’, formulation containing pyrethrin active ingredients) increased phenotypic resistance to streptomycin and ciprofloxacin in single species experiments with *E. coli*^101^. The organophosphate insecticide chlorpyrifos also increased ARGs and *intI1* in bulk soil, but not rhizosphere soil, when tested in microcosms followed by qPCR^112^. The authors speculated that the difference between the soils could be due to reduced availability of the insecticide in the two soil types, or differences in bacterial species present, highlighting how bioavailability (mentioned previously with regards to metals) is also a concern for other non-antibiotic agents.

Furthermore, studies have shown correlations between tolerance to insecticides and antibiotic resistance in isolates obtained from contaminated fields^113–115^. Rangasamy et al., demonstrated that *Bacillus* strains isolated from soils contaminated with lindane, α-endosulfan, and β-endosulfan insecticides, displayed phenotypic resistance to ampicillin, cefotaxime, chloramphenicol, streptomycin, and tetracycline, however, there was no comparison to control isolates^113^. Additionally, removal of a plasmid with insecticide degrading properties from these *Bacillus* strains increased their susceptibility to the antibiotics, suggesting a link between insecticide degradation genes and antibiotic resistance or degradation^113^. Similarly, isolates obtained from fields contaminated with unspecified pesticides were found to be phenotypically tolerant to insecticides, fungicides, and antibiotics, with isolates often containing *ampC, tetM, ermD, mecA,* and *ermG*, genes^115^. However, these results are correlative, and environmental isolates in such studies are often exposed to a myriad of stressors that may increase resistance evolution and HGT, so it may not be the sole action of the insecticide that resulted in antibiotic resistance.

To conclude, research into co-selection for antibiotic resistance by PPPs is in its early stages, with many of the studies showing evidence of co-selective effects, yet others show varied results. In addition to herbicides, fungicides, and insecticides, there are other groups of PPPs including molluscicides, acaricides, rodenticides, and plant growth regulators. To our knowledge, there are no studies on these classes of PPPs and co-selection for antibiotic resistance.

Furthermore, the active ingredients of PPPs are often applied alongside other ingredients as part of a chemical formulation, which improve the efficacy of the active chemical (i.e., adjuvants, which act to increase surface area and have surface active properties). Therefore, large quantities of adjuvants, solvents, and other ingredients may also be applied to crops and soils and should be investigated for their co-selective potential. The 2017 study by Kurenbach et al., did investigate some common herbicide co-formulants (‘Pulse Penetrant carboxymethyl cellulose’ and ‘Tween80’) for their co-selective potential. They found varied effects on phenotypic resistance that were dependent on species, antibiotic of interest, and co-formulant^104^.

## Knowledge gaps

There is clear evidence for co-selection by various non-antibiotic agents as summarised in this review (Table 1), and the potential for co-selection by others which have shown varied results, but there are still significant knowledge gaps, including data specifically using environmentally relevant concentrations, species, or microbial communities. Particularly, there is limited research on co-selection for antibiotic resistance by NADs and PPPs and key knowledge gaps are discussed below. A theme common to all the recommendations below, is that despite there being strong evidence for co-selection in many of the studies presented, the underlying co-selection mechanisms remain unclear. Further studies should include experiments that specifically confirm the mechanism of co-selection e.g., co-resistance, cross-resistance, co-regulation, or any combination of these, where possible. This may not always be feasible, if for example, a multidrug efflux pump (cross-resistance) was located on a plasmid with an antibiotic resistance gene (co-resistance), with the expression of both genes under control of the same promoter (co-regulation). No one mechanism could be ascribed to co-selection in this case. However, for simpler studies with less genetic complexity, the co-selection mechanism may be easier to identify. This would be beneficial to not only improve the robustness of results, but also to begin to discern the most common/concerning mechanism of co-selection, which could direct future studies.

**Table 1:** Overview of the design and outcome of the studies presented in this review

Non-antibiotic agent	Single or mixture	Co-selection mechanism	Observation	Experimental Design	References
Metal	NA	Co-resistance	Co-existence of metal and antibiotic resistance genes on plasmids	Genomic	Li et al. ^40^
Metal	NA	Co-resistance	Co-existence of metal and antibiotic resistance genes on plasmids	Genomic	Pal et al. ^10^
Metal	Single and mixture	Co-resistance	Selection for resistance plasmid carrying metal and antibiotic resistance	Laboratory microcosm, single species	Gullberg et al. ^47^
Metal	Single	Cross-resistance	Efflux pump removes metals and antibiotics from cell	Laboratory microcosm, single species	Adhikary et al. ^41^
Metal	Single	Co-regulation	Upregulation of resistance genes to metals and antibiotics	Laboratory microcosm, single species	Lee et al. ^42^
Metal	Single and mixture	Unconfirmed co-selection mechanism	Increase in phenotypic resistance	Laboratory microcosm, single species	Chen et al. ^48^
Metal	Single	Unconfirmed co-selection mechanism	Increase mutation rate, enriched for de-novo mutants with resistance	Laboratory microcosm, single species	Li et al. ^49^
Metal	Single and mixture	Unconfirmed co-selection mechanism	Increase mutation rate	Laboratory microcosm, single species	Li et al. ^50^
Metal	Single	Unconfirmed co-selection mechanism	Increase in HGT of ARGs	Laboratory microcosm, single species	Zhang et al. ^51^
Metal	Single and mixture	Unconfirmed co-selection mechanism	Increase in phenotypic resistance	Laboratory microcosm, single species	Jun et al. ^101^
Biocide	Single	NA	No increase in antibiotic resistance	Laboratory, microcosm, single species	Beier et al. ^56^
Biocide	Single	Unconfirmed co-selection mechanism	Upregulation of ARGs, increased antibiotic resistance	Laboratory microcosm single species	Wand et al. ^57^
Biocide	NA	Co-resistance	Co-existence of biocide, and antibiotic resistance genes	Genomic	Pal et al. ^10^
Biocide	Single	Cross-Resistance	Increased resistance to biocides and antibiotics	Laboratory, microcosm community	Tandukar, ^60^
Biocide	Single	Unconfirmed co-selection mechanism	Increased mutation rates, changes to gene expression	Laboratory, microcosm single species	Lu et al. ^61^
Biocide	Single	NA	Decreased abundance of ARGs and metal and biocide resistant genes	Laboratory, microcosm community	Murray et al. ^63^
Biocide	Single	Unconfirmed co-selection mechanism	Increased phenotypic resistance	Laboratory microcosm, single species	Li et al. ^62^
Non-antibiotic drug	Single	NA	Effects on growth	Laboratory microcosm single species	Sud and Feingold. ^69^
Non-antibiotic drug	Single	NA	Effects on growth	Laboratory microcosm single species	Alsterholm et al. ^68^
Non-antibiotic drug	Single	Unconfirmated co-selection mechanism	Effects on growth, changes to proteome and bacterial diversity	Laboratory microcosm community	Li et al. ^70^
Non-antibiotic drug	Single	NA	Effects on growth	Laboratory microcosm single species	Younis et al. ^72^
Non-antibiotic drug	Single	Cross resistance	Effects on growth. *TolC* knockout affected growth	Laboratory microcosm single species	Maier et al. ^71^
Non-antibiotic drug	Single	NA	Effects on growth	Laboratory microcosm, single species	Campbell et al. ^74^
Non-antibiotic drug	Single	Unconfirmed co-selection mechanism	Increased mutation rate, gene expression changes	Laboratory microcosm, single species	Jin et al. ^80^
Non-antibiotic drug	Single	Unconfirmed co-selection mechanism	Increased horizontal gene transfer	Laboratory microcosm, single species	Wang et al. ^75^
Non-antibiotic drug	Single	NA	Increased horizontal gene transfer	Laboratory microcosm, single species	Wang et al. ^78^
Non-antibiotic drug	Single	Unconfirmed co-selection mechanism	Increased horizontal gene transfer	Laboratory microcosm, single species	Jia et al. ^76^
Non-antibiotic drug	Single	Unconfirmed co-selection mechanism	Increased horizontal gene transfer	Laboratory microcosm, single species	Ding et al. ^77^
Non-antibiotic drug	Single	NA	Decreased horizontal gene transfer	Laboratory microcosm, single species	Buckner et al. ^79^
Non-antibiotic drug	Single	Unconfirmed co-selection mechanism	Increased phenotypic resistance	Laboratory microcosm, single species	Guðmundsdóttir et al. ^81^
Non-antibiotic drug	Single	Unconfirmed co-selection mechanism	Increased mutation rates and selection for antibiotic resistant mutants	Laboratory microcosm, single species	Meunier et al. ^82^
Non-antibiotic drug	Single	Unconfirmed co-selection mechanism	Altered gene expression, increased phenotypic resistance	Laboratory microcosm, single species	Riordan et al. ^83^
Non-antibiotic drug	Single	Unconfirmed co-selection mechanism	Altered gene expression, increased phenotypic resistance	Laboratory microcosm, single species	Kyono et al. ^84^
Non-antibiotic drug	Single	NA (potential cross-resistance)	Substrate of efflux pumps	Laboratory microcosm, single species	Laudy et al. ^86^
Non-antibiotic drug	Single	NA	No increase in phenotypic resistance, or co-selection effects, but altered gene expression	Laboratory microcosm, single species	Hall et al. ^88^
Non-antibiotic drug	Mixture	Unconfirmed co-selection mechanism	Increased antibiotic resistance in mixture	Laboratory microcosm, single species	Shi et al. ^30^
PPP (Mixture)	Mixture	Unconfirmed co-selection mechanism	Increased phenotypic and genetic resistance to 1 of 4 tested antibiotics (streptomycin)	Laboratory microcosm, single species	
PPP (Herbicide)	Single	Unconfirmed co-selection mechanism	Increase in ARGs	Laboratory microcosm, soil community	Liao et al. ^98^
PPP (Herbicide)	Single and mixture	Unconfirmed co-selection mechanism	Increase in phenotypic resistance	Laboratory microcosm, single species	Jun et al. ^101^
PPP (Herbicide)	Single	Unconfirmed co-selection mechanism	Changes in phenotypic resistance	Laboratory microcosm, single species	Kurenbach et al. ^104^
PPP (Herbicide)	Single and mixture	Unconfirmed co-selection mechanism	Changes in phenotypic resistance. Changes in resistance evolution rate	Laboratory microcosm, single species	Kurenbach et al. ^103^
PPP (Herbicide)	Single	Unconfirmed co-selection mechanism	Changes in phenotypic resistance	Laboratory microcosm, single species	Kurenbach et al. ^102^
PPP (Herbicide)	Single	Unconfirmed co-selection mechanism	Increase in resistance evolution	Laboratory microcosm, single species	Braz et al. ^100^
PPP (Herbicide)	Single	Unconfirmed co-selection mechanism	Increase in horizontal gene transfer	Laboratory microcosm, single species	Zhang et al. ^99^
PPP (Fungicide)	Single	Unconfirmed co-selection mechanism	Increase in ARGs	Soil microcosm, community	Zhang et al. ^105^
PPP (Fungicide)	Single	Unconfirmed co-selection mechanism	Increase in phenotypic resistance	Soil mesocosm, community	Aleksova et al. ^107^
PPP (Fungicide)	Single	Unconfirmed co-selection mechanism	Increase in phenotypic resistance	Soil mesocosm, community	Aleksova et al. ^108^
PPP (Fungicide)	Single	Unconfirmed co-selection mechanism	Increase in ARGs	Laboratory soil microcosm and worm gut, communities	Zhang et al. ^109^
PPP (Fungicide)	Single	Unconfirmed co-selection mechanism	Increased horizontal gene transfer	Laboratory, single species, and two species mixtures	Song et al. ^110^
PPP (Fungicide)	Single	Unconfirmed co-selection mechanism	Increased horizontal gene transfer	Laboratory, single species and two species mixtures	Zhang et al. ^111^
PPP (Insecticide)	Single	Unconfirmed co-selection mechanism	Increase in ARGs	Laboratory microcosm, community	Guo et al. ^112^
PPP (Insecticide)	Single	NA	Presence of resistant bacteria from contaminated environments	Environmental samples	Nawab et al. ^114^
PPP (Insecticide)	Single	NA	Presence of resistant bacteria from contaminated environments	Environmental samples	Anjum et al. ^115^
PPP (Insecticide)	Single	NA	Presence of resistant bacteria from contaminated environments	Environmental samples	Rangasamy et al. ^113^
PPP (Insecticide)	Single and mixture	Unconfirmed co-selection mechanism	Increase in phenotypic resistance	Laboratory microcosm, single species	Jun et al. ^101^
PPP (Co-formulants)	Single	Unconfirmed co-selection mechanism	Increase in phenotypic resistance	Laboratory microcosm, single species	Kurenbach et al. ^104^

## Where does co-selection occur?

Although co-selection has been shown to occur in laboratory experiments, the extent and implications of co-selection are largely unknown. For example, in which bacterial microbiomes can co-selection occur? Further research should aim to identify these, so their study can be prioritised. This could be addressed by testing a wider range of matrices (e.g., sewage/river sediment/soil/surface water communities, in addition to laboratory strains).

Furthermore, it is important to consider the vast number of agents or mixtures already in use and consider the future development of new agents or mixtures. Testing of new agents is clearly recognised to be important but re-testing of agents previously not considered to have co-selective effects may also be necessary. For example, some NADs are now being recognised as having antibacterial effects, but may also have co-selective effects, despite being in clinical use for decades^81,116^. In addition, a better understanding of the usage of non-antibiotic agents would help to determine hotspots and mitigation strategies. This is easier for some non-antibiotic agents than others. Prescription records are particularly well developed for NADs, but over-the-counter use is less well monitored. Usage of other non-antibiotic agents such as biocides and PPPs face more challenges in monitoring. For example, pesticide active ingredients are present on their own, and in combination with other active ingredients in a wide range of commercial products, which are often easily purchased over the counter, making monitoring of use difficult. Many of the decisions as to how, when, and where the products will be used (within the advised restrictions) are made by the consumer. Only when we have a full understanding of where each agent acts as a co-selecting agent can we implement mitigation strategies.

## At which concentrations does co-selection occur?

Following the identification of co-selective agents, it is important to investigate the concentration ranges capable of selecting for antibiotic resistance. Many non-antibiotic agents will be present in different settings at a range of concentrations, for example, as residues on surfaces, and in the environment as diluted contaminants. More studies are needed to understand potential selective hotspots, especially for compounds that are applied at high concentrations (such as biocides and PPPs). However, as detailed in the introduction, there has been increasing interest in selection for ARGs by antibiotics at sub-MIC concentrations, and a growing understanding that this increases the window of selection or persistence of resistance mechanisms in bacterial communities^19,20^. Worryingly, lower selective pressures are more likely to result in selection and persistence of resistance genes with a low fitness cost to the bacterium. This means they may persist in the absence of selection far longer than ARGs with higher fitness cost, which would only be selected for by higher concentrations of co-selective agents^117–119^. Some research has already demonstrated that certain non-antibiotic agents are capable of a similar effect, increasing resistance at sub-inhibitory concentrations^47,49^. There needs to be focused study of the co-selective windows for other non-antibiotic agents, as without data it is impossible to pinpoint where and when there could be fixation and dissemination of ARGs, and transfer to opportunistic pathogens resulting from co-selective agent contamination.

## Appreciating chemical complexities

It is of paramount importance to appreciate chemical complexities, including their potency and behaviour that is driven by ever-changing natural environments. Experimental designs on co-selection studies tend to focus on individual chemicals at certain concentration levels. The assumption is made that these chemicals are constant in the context of dynamic changes in microbial communities, however, this is not the case. Chemicals are in a dynamic equilibrium between physical and biological environments. They also continuously partition between liquid and solid interfaces (e.g., suspended particulate matter or sediments) and this affects their bioavailability. In general, chemicals with lower polarity (higher hydrophobicity, usually described by logKow or logP constants) tend to be more persistent and bioaccumulate in biota and sediments. The potency and bioavailability of chemicals are also directly linked with their state-of-charge. Chemicals can be either non-charged (neutral, e.g., pure water), positively/negatively charged (e.g., negatively charged anti-inflammatory drugs or positively charged beta-blockers), or zwitterionic (e.g., quinolone antibiotics) in their surrounding environment, and their charge will be driven by changing pH. The pH of natural environments can change significantly from neutral to acidic which might be a result of natural processes as well as contamination, e.g., acid mine drainage. This change in pH might have an impact on chemicals in question. As an example: ibuprofen with pKa (acid-base constant) of 4.85 will be largely protonated in a heavily polluted environment (e.g., rivers affected by acid mine discharges), but it will deprotonate (to gain negative charge) in a neutral, healthy environment. This change of charge state has critical consequence to its bioavailability, potency, and transport across the cell wall. Large scale monitoring campaigns clearly show variable presence of chemicals that is driven not only by usage but also by their properties^120^, e.g., plant uptake is to a large extent driven by hydrophilicity and charge^121^. Chemicals, when they pass through human/animal bodies, are subject to metabolism that leads to the formation of metabolites. Phase II metabolites such as glucuronides, are relatively unstable in wastewater and are subject to microbial cleavage of parent compounds^122^. This is of key consequence to risk assessment of chemicals in the environment, as it is commonly assumed that metabolites are less potent than parent chemicals^123^. Lastly, many chemicals (including most antibiotics) have stereocenters (e.g., chiral centres) which enables them to exist in several configurational forms, each potentially triggering different biological responses^124^.

The complexities of chemicals, and chemical metabolites, is an important consideration in their potential co-selective effects, and the extent to these effects in different environments. For example, a wastewater treatment plant output to the freshwater system will be dispensing parent compound, metabolites, and several forms of these compounds into an environment that could be changeable in terms of pH, and the bacteria that are present there, so the effects of this input to the freshwater system may vary over season/time. Further work should try to understand and incorporate some of this complexity into experimental design to determine how important these chemical properties are in co-selection.

## What are the co-selective effects in complex (microbial and chemical) contexts?

Most research published to date has focused on the incidence of co-selection at the single species level. However, bacteria exist in the environment within complex microbial communities. Previous research has shown that selection for antibiotic resistance by some aminoglycoside antibiotics is reduced when a bacterium is in a community, compared to when grown in isolation^125^. Similar effects could occur with exposure to non-antibiotics in complex communities, but this is yet to be researched. Additionally, when considering the effects of selective agents on microbial communities, research should consider that different microbiomes exist in different environments and may respond differently to the same selective pressure.

Similarly, these agents are rarely present in isolation. In environmental compartments e.g., wastewater and soils, antibiotics are found alongside metals, biocides, NADs, and other potentially selective agents. However, the research literature on the selective effects of antibiotic mixtures, and of antibiotics alongside non-antibiotic agents, is limited. MICs and MSCs are usually experimentally determined using single species models, and most often for single antibiotic stressors^18,126,127^. Therefore, it is likely that combined exposure risks (of multiple agents) are underappreciated^30^. Mixture effects can be both positive (additive or synergistic), or negative (antagonistic). There is some evidence that suggests particular combinations of agents, such as metals and antibiotics, or antibiotics alongside NADs, can increase antibiotic resistance. For example, it was shown that the combined exposure of *E. coli* to duloxetine (an antidepressant) and chloramphenicol increased the transcription of ARGs compared to exposure to each compound alone^119^. Interactions may be agent specific, for example, a tetracycline and arsenic mixture decreased the MSC of tetracycline^47^, whereas the MSC of ciprofloxacin increased in the presence of zinc^128^.

Not only are bacterial communities exposed to a mixture of agents, but those agents degrade at different rates, and into a variety of different metabolites. This results in the communities being exposed to various metabolites at variable concentrations, in addition to parent compounds, that could have co-selective effects^129,130^. Future research should aim to address this, by exposing bacterial communities present in ecological compartments (contaminated and pristine soil environments, the aquatic environment, marine, the gut, clinical environments, veterinary environments, wastewater etc.) to combinations of antibiotic and non-antibiotic agents and context-relevant metabolites.

## Is there evidence of increased resistance to non-antibiotic agents, after exposure to antibiotics?

Research concerning increased resistance to antibiotics after exposure to non-antibiotics is increasing, but research into the reverse is lacking. However, there is some evidence that antibiotics can co-select for non-antibiotic agent resistance. After adaptation to erythromycin, *E. coli* demonstrated increased resistance to the biocide triclosan^131^. Conversely, adaptation to colistin has been shown to have no effect on cross-resistance to chlorhexidine^57^. Efflux pumps may be important in this process. For example, the AcrAB-TolC efflux pump can provide resistance to a range of antibiotics^132^ and remove biocides from within the cell^28^. It could be argued that after exposure to antibiotics, these broad range resistance mechanisms may also confer resistance to biocides. Despite this, there is a lack of understanding of this phenomenon, which might be a concern for hospitals and other clinical settings since disinfectant biocide use, and even NAD use, could lead to increased antibiotic resistant nosocomial infections. Future studies should test a range of concentrations of these agents and use bacterial communities that are representative of the various compartments that might be affected.

## The problem of bias in gene databases

ARG databases contain genes mostly identified in clinical isolates after exposure to antibiotics. It is likely that non-antibiotic agents could be selecting for novel or as-yet unannotated resistance genes, which are therefore not detected in current studies. Novel genes could be of great concern, particularly if there is a high risk of transfer of these novel genes into human, animal, or plant pathogens. Methods such as functional metagenomics, and predictive methods (e.g., machine learning^133^), alongside confirmatory experimental work could be used to identify these novel genes^134^.

## Challenges for risk management

Current environmental risk assessments for antibiotics do not consider the risk of selection for antibiotic resistance in the environment. The only tests on microorganisms focus on aerobic capacity and community functioning, and are tested on algae^96^, or on activated sludge communities^97^. Recent research calls for the inclusion of risk of selection for resistance^126^. Although the inclusion of this in environmental risk assessment would be a positive step towards risk mitigation, it is important to also consider the co-selective properties of non-antibiotic agents. Simply addressing the risk posed by antibiotics may not be sufficient to remove the selective pressure if co-selective agents are present in the environment. Mixture effects are also not routinely identified in terms of environmental risk, and therefore incorporating these into environmental risk assessment would be a step forward in protecting the environment in terms of selection, maintenance and dissemination of ARGs and resistant bacteria.

## Conclusions

Due to the widespread presence of potentially co-selective agents, and in an ever increasingly connected world, reducing antibiotic usage to tackle antibiotic resistance may not have the desired effects if no mitigations are put in place to reduce co-selective agents. There are around 355,000 chemicals or mixtures of chemicals thought to be in use^135^. Anthropogenic production of these chemicals has led to some considering that we have “crossed a planetary boundary” for pollution by novel entities meaning that we are “outside of the safe operating space” for chemical pollution^136^. The number of chemicals and agents that have been tested for their co-selective effects is merely a fraction of this total number. Even among those that have been tested (e.g., PPPs), their co-selective potential clearly varies under different contexts and more research is required to fully understand the threat that these agents, and mixtures of agents may have in terms of AMR. Our understanding of the individual and mixture effects of agents and their behaviour in different environments is also poor, and so we may be underestimating their effects. The One Health perspective highlights the connectivity between different environmental compartments (e.g., between human and veterinary health, the soil, and the aquatic environment). When considering this, and the vast numbers of agents that are present globally, there is likely no area immune to the co-selective effects of agents that can select for antibiotic resistance.
